# 

**Published:** 1850-02

**Authors:** 


### CONTENTS

OF NO. II.

### ORIGINAL COMMUNICATIONS.

ESSAYS AND CASES.

ART. I.—The Bandage in the Treatment of Fractures, Ulcers, Gun-Shot Wounds, Hemorrhage, &c., &c. By THEODORE S. BELL, M. D., of Louisville, Ky. - - 93

ART. II.—A Case of Rupture of the Ilium and Omentum, and fatal result in twelve hours, By F. H. GORDON, M. D. 142

REVIEWS.

ART. III.—A Critical Analysis of the "Review of Professor B. W. Dudley's article on Aneurism, in the November No. of the Louisville Medical Journal. Transylvania Medical Journal, Vol. 1, No. III. - - - - 144

ART. IV.—Lectures on the Diseases of Infancy and Childhood. By CHARLES WEST, M. D., F. R. C.; Physician to the Royal Infirmary for Children; Physician Accoucheur to the Middlesex Hospital; and Lecturer on Midwifery at St. Bartholomew's Hospital.

On the Diseases of Children. By FLEETWOOD CHURCHILL, M. D., M. R. I. A., Hon. Fellow of the College of Physicians, Ireland; Hon. Member of the Philadelphia Medical Society, etc., etc., author of "The Theory and Practice of Midwifery," "On the Diseases of Females," etc., etc.

A Practical Treatise on the Diseases of Children. By D. FRANCIS CONDIE, M. D., Sec. of the College of Physicians; Member of the American Medical Association, &c., &c. - - - - - - - 163

ART. V.—Principles of Human Physiology; with their Chief Applications to Pathology, Hygiene, and Forensic Medicine. By William B. Carpenter, M. D., F. R. S., F. G. S., Examiner in Physiology in the University of London; Lecturer on Physiology at the London Hospital Medical School, - - - - - - 168

Art. VI.—The Transactions of the American Medical Association. Instituted 1847. Vol. II. - - - 174

ORIGINAL INTELLIGENCE.

The Reviewer of Professor Dudley's Essays, - - 178
Kentucky Surgery, - - - - - 178
American Surgery, - - - - - - 179
Medical Men's Per-Centage on Prescriptions, - - - 181
The Medical Society of Montgomery County, Tenn. - 182
Typhoid Fever in Alabama, - - - - - 182
Postage on Scientific Journals, - - - - 183
The Medical Journal for March, - - - 184

